# Letter to the editor of implementation science in response to “Implementation Science in maternity care, A scoping Review” by Dadich, Piper, and Coates (2021)

**DOI:** 10.1186/s13012-021-01129-9

**Published:** 2021-08-16

**Authors:** Rachel Blankstein Breman, Rebecca Feldman Hamm, Jennifer A. Callaghan-Koru

**Affiliations:** 1 Department of Partnerships, Professional Education and Practice, School of Nursing, University of Maryland, Baltimore, MD USA; 2 Department of Obstetrics and Gynecology, University of Pennsylvania Perelman School of Medicine, Philadelphia, USA; 3 Department of Sociology, Anthropology and Public Health, University of Maryland, Baltimore County, Baltimore, USA

Dear Editors:

Thank you for the recent publication “Implementation Science in maternity care, A scoping Review” by Dadich, Piper, and Coates. As the authors note, preventable maternal morbidity and mortality is an urgent health crisis. Maternity care is plagued by evidence-based practice gaps at two extremes—“too much too soon” and “too little too late.” In both high- and low-resource settings, there is underuse of many effective interventions, as well as unwarranted over intervention in normal physiologic labor [1]. As this review demonstrates, maternity care is in great need of implementation research to close these gaps. While we applaud Dadich and colleagues’ attention to this important issue, we also have concerns about the study’s methodology. Specifically, we believe the authors should have used broader search terms in order to provide a comprehensive assessment of implementation science in maternity care.

The methods state that the search terms were limited to two—“implementation” and “maternity.” Restricting the search to articles with the exact term “maternity” provides a vastly incomplete picture of the literature surrounding maternity care. Maternity care is a continuum, encompassing prenatal, intrapartum, and postpartum care, and involves a mix of providers, including midwives, obstetricians, maternal-fetal medicine specialists, and nurses. Search terms essential to any review regarding maternity care would include perinatal, prenatal/antenatal, labor, intrapartum, obstetric, midwifery, birth, childbirth, postpartum, and maternal. In order to fully understand implementation research within maternity care, we need to consider the different, but related terms that can be employed across studies and settings. We do not consider these terms to be “euphemisms,” as suggested in the paper’s discussion, but rather critical terms to identify relevant literature within the scope of this review.

A prior systematic search we conducted with broader maternal health search terms identified 58 additional implementation research studies addressing maternity care for the United States alone. We are specifically concerned with the absence of important implementation research studies on critical interventions for the reduction of maternal morbidity and mortality globally:

- The World Health Organization’s Safe Childbirth Checklist includes interventions to prevention of morbidity due to hypertension, hemorrhage, infection, and other causes. The BetterBirth trial, a large trial implementing the checklist in India, published multiple reports not captured in the review [2–5]. Implementation of this checklist, with coaching support, yielded equivocal results, an important consideration for future implementation initiatives and research in low-resource setting studies.
- Postpartum health and monitoring are also an essential aspect of maternity care. One example is a shelf-stable, relatively inexpensive injection of oxytocin in the third stage of labor to prevent postpartum hemorrhage and death. This practice is frequently referred to as active management of the third stage of labor (AMTSL) [6]. Multiple studies have assessed implementation of AMTSL [7–9], yet none of these or any on this topic were included in the review.
- Overuse of cesarean and underutilization of vaginal birth after cesarean (VBAC) have contributed to maternal morbidity and mortality in more developed countries [10, 11]. Several studies addressing implementation of strategies and practices to prevent primary cesarean birth in countries where overuse is a major challenge to improve birth outcomes were not included in this review [12–14].

We also note the exclusion of published studies utilizing implementation research frameworks and theories in maternity care. We are aware of nine additional studies employing the CFIR in maternity care published during the search period within the USA [15–23]. These studies took place in hospital labor and delivery units, outpatient prenatal/postpartum clinics, and community organizations, and addressed implementation of practices related oral health, obstetric hemorrhage, and long acting reversible contraception, among other interventions. Similar studies may have been overlooked from other countries.

The breadth of maternity care settings, and diversity of implementation constraints between settings, makes it challenging to map this literature in one review paper. Dadich and colleagues, like implementation scientists assessing literature in other clinical areas [24], rightly note the need to promote the consistent application of implementation science theories and frameworks to develop applicable knowledge about how to best support implementation across maternity care settings. We would also advocate for the need to adapt and apply implementation science measures to maternity care. Efforts to synthesize findings across studies that use the same frameworks, theories, and/or measures will be a great asset in advancing implementation science for maternity care. While there remains much work to be done to support implementation in maternity care, ensuring that we learn from all prior research will allow us to more effectively and efficiently target future efforts for the greatest benefit of maternity patients.

## Data Availability

N/A
